# Impact of nutrition on skin wound healing and aesthetic outcomes: A comprehensive narrative review

**DOI:** 10.1016/j.jpra.2024.01.006

**Published:** 2024-01-23

**Authors:** Ishith Seth, Bryan Lim, Jevan Cevik, Dylan Gracias, Marcel Chua, Peter Sinkjaer Kenney, Warren M. Rozen, Roberto Cuomo

**Affiliations:** a Department of Plastic Surgery, Peninsula Health, Melbourne, Victoria 3199, Australia; b Central Clinical School at Monash University, The Alfred Centre, 99 Commercial Rd, Melbourne, Victoria 3004, Australia; c Department of Plastic Surgery, Odense University Hospital, J. B. Winsløwsvej 4, Odense 5000, Denmark; d Department of Plastic and Breast Surgery, Aarhus University Hospital, Aarhus 8200, Denmark; e Plastic Surgery Unit, Department of Medicine, Surgery and Neuroscience, University of Siena, Siena 53100, Italy

**Keywords:** Nutrition, Skin healing, Wound healing, Aesthetic, Cosmetic outcomes

## Abstract

**Background:**

While current wound treatment strategies often focus on antimicrobials and topical agents, the role of nutrition in wound healing and aesthetic outcomes is crucial but frequently overlooked. This review assesses the impact of specific nutrients and preoperative nutritional status on surgical outcomes.

**Methods:**

A comprehensive search was conducted in PubMed, Scopus, Web of Science, and the Cochrane Library, from the inception of the study to October 2023. The study focused on the influence of macronutrients and micronutrients on aesthetic outcomes, the optimization of preoperative nutritional status, and the association between nutritional status and postoperative complications. Inclusion criteria were English language peer-reviewed articles, systematic reviews, meta-analyses, and clinical trials related to the impact of nutrition on skin wound healing and aesthetic outcomes. Exclusion criteria included non-English publications, non-peer-reviewed articles, opinion pieces, and animal studies.

**Results:**

Omega-3 fatty acids and specific amino acids were linked to enhanced wound-healing and immune function. Vitamins A, B, and C and zinc positively influenced healing stages, while vitamin E showed variable results. Polyphenolic compounds showed anti-inflammatory effects beneficial for recovery. Malnutrition was associated with increased postoperative complications and infections, whereas preoperative nutritional support correlated with reduced hospital stays and complications.

**Conclusion:**

Personalized nutritional plans are essential in surgical care, particularly for enhanced recovery after surgery protocols. Despite the demonstrated benefits of certain nutrients, gaps in research, particularly regarding elements such as iron, necessitate further studies. Nutritional assessments and interventions are vital for optimal preoperative care, underscoring the need for more comprehensive guidelines and research in nutritional management for surgical patients.

## Introduction

**Figure 1:**
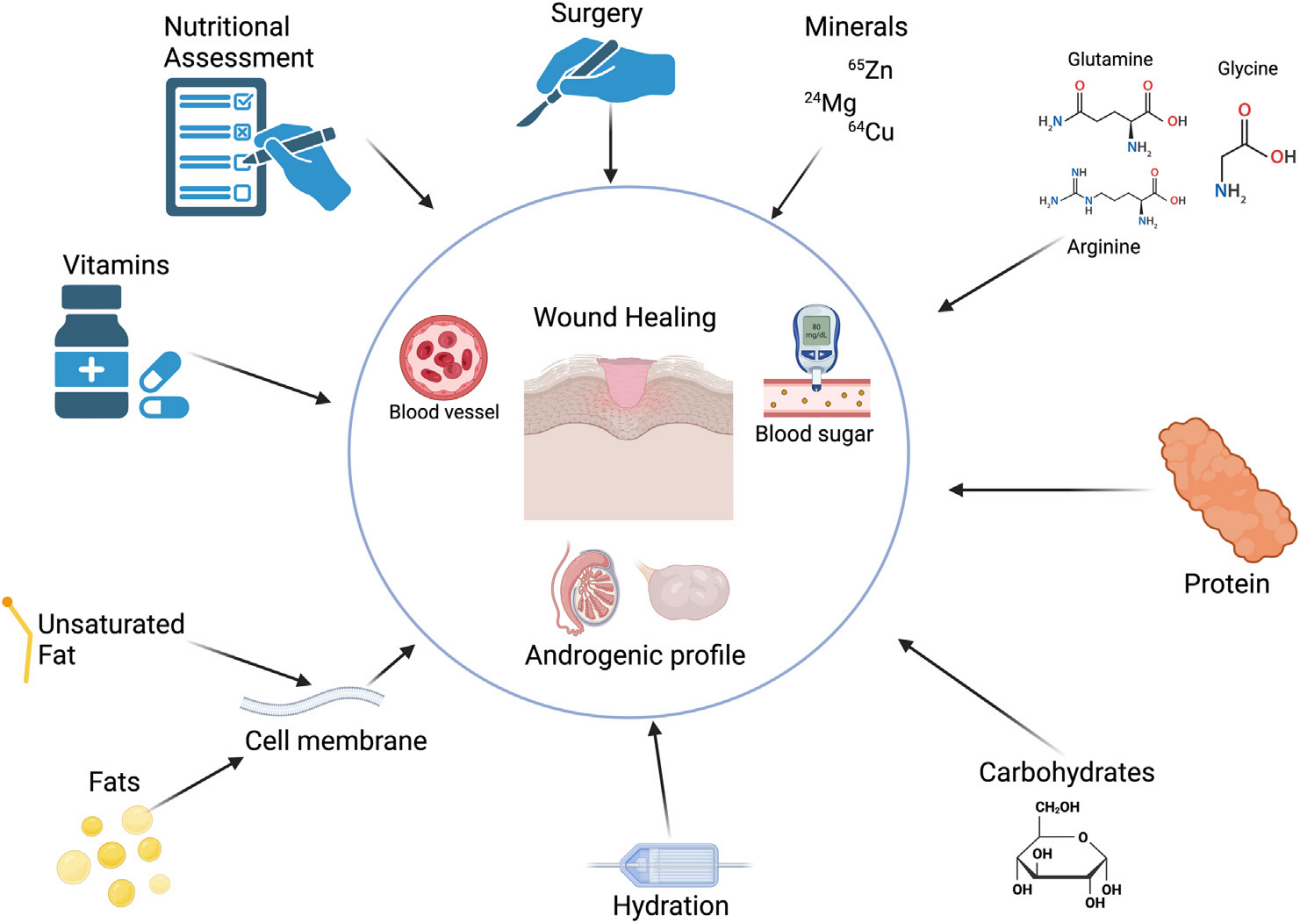
Factors that potentially influence wound healing and aesthetic outcomes.

Wound healing is an energy-intensive process, requiring an array of macronutrients and micronutrients to restore skin integrity efficiently.^1^ Macronutrients, including carbohydrates, fats, proteins, and fluids, along with micronutrients, such as vitamins and minerals, collectively orchestrate the seamless progression of wound healing (Figure 1).^2^ The caloric demands for protein synthesis, a cornerstone in forming granulation tissue,^3^ underscore the heightened nutritional requirements during the reparative phases.^4^ The impact of nutrition on the aesthetic outcomes of wound healing is substantial, an appropriate supply of nutrients is crucial for reducing scar formation and supporting the intricate process of skin remodeling. While minor wounds may not significantly tax the nutritional reserves of the body, larger wounds, particularly extensive thermal burns, can precipitate a considerable nutritional deficit, further compounded by perioperative fasting protocols that may disrupt the timely resumption of diet that is critical for recovery.^5^, ^6^, ^7^

Tight glycemic regulation is a crucial aspect in optimizing wound healing, with uncontrolled hyperglycemia known to impede fibroblast and endothelial cell functions, particularly in patients with diabetes.^8^ The historical context of vitamins, such as vitamin C and its association with scurvy, elucidates their role as a co-substrate for hydroxylase enzymes imperative for collagen synthesis^9^. Similarly, vitamin A and zinc have been recognized for their contributions to epithelial growth, angiogenesis, collagen synthesis, wound strength, and epithelization. Yet, the efficacy of supplementation in patients with apparent nutritional deficiencies remains contentious.

Beyond nutritional elements, lifestyle factors, such as smoking and alcohol consumption, are increasingly being acknowledged for their detrimental effects on wound healing.^10^ Smoking with its plethora of harmful substances, notably nicotine, induces vasoconstriction and disrupts microcirculation, thereby impairing wound healing.^10^ The inhibition of cellular migration and neutrophil activity during the inflammatory phase further exacerbates wound complications in smokers compared with that in nonsmokers. Likewise, the consumption of alcohol, including chronic abuse and acute intoxication, has been linked to an elevated incidence of surgical wound infections and impaired wound healing.^10^

This comprehensive narrative review aims to dissect the complexities of nutritional contributions to skin wound healing and their subsequent impact on aesthetic outcomes. By amalgamating existing scientific literature with novel insights, we aspire to furnish a detailed narrative that elucidates the multifaceted interplay between nutrition, lifestyle factors, and skin repair mechanisms. The objective is to enlighten medical practitioners, especially in the field of aesthetic medicine, with an evidence-based framework to optimize nutritional strategies, enhance surgical outcomes, and promote efficacious wound healing.

## Methods

PubMed, Web of Science, EMBASE, Scopus, and the Cochrane Library databases were searched from the inception of this study until October 2023 by two independent authors (IS and BL). The search keywords and phrases used were “nutrition,” “wound healing,” “skin,” “aesthetic outcomes,” “diet,” “macronutrients,” “micronutrients,” “vitamins,” “minerals,” “smoking,” and “alcohol.” References of previous reviews and included studies were screened for any additional studies. The inclusion criteria consisted of English language articles, peer-reviewed original research articles, systematic reviews, meta-analyses, clinical trials, studies that specifically address the impact of nutrition (macronutrients, micronutrients, and supplementation) on skin wound healing, studies that investigate the role of lifestyle factors (smoking, alcohol) on wound healing and aesthetic outcomes, and studies involving human participants of any age, sex, and ethnicity. The exclusion criteria were articles not published in English, non-peer-reviewed literature, opinion pieces, editorials, letters to the editor, studies not directly related to nutrition and skin wound healing or aesthetic outcomes, and animal model studies.

Owing to the nature of this study's design (narrative review), ethical approval was not required. Academy of Nutrition and Dietetics narrative review checklist was strictly adhered to (https://www.elsevier.com/__data/promis_misc/ANDJ%20Narrative%20Review%20Checklist.pdf).

## Results

### Essential nutrients for patients undergoing cosmetic treatments

Nutritional status, a product of nutrient intake, absorption, and utilization, holds a pivotal role in an individual's overall health. A robust link exists between well-being and adherence to a balanced diet, an understanding that has been well-established in contemporary research.^11^^,^^12^ Recognizing this, the early involvement of dietary teams alongside the surgical team is crucial to optimize nutritional care for pre-surgical patients to optimize their surgical course and postoperative recovery.^13^ This principle extends to those undergoing minimally invasive and aesthetic procedures, emphasizing the importance of nutritional screening and assessments to preemptively identify, address, and prevent malnutrition of any form prior to surgery.^14^, ^15^, ^16^ Ensuring an adequate intake of all essential nutrients is therefore paramount to enhance the outcomes of aesthetic interventions.^3^^,^^10^^,^^17^, ^18^, ^19^, ^20^ Carbohydrates, fats, proteins, and fluids are crucial macronutrients that cater to the heightened energy demands during the wound-healing process. Wound healing is an energy-intensive process, with caloric needs during this period estimated at 30–35 kcal/kg, or up to 40 kcal/kg for underweight individuals.^21^^,^^22^ These requirements may vary based on factors such as age, comorbidities, body weight, activity level, and the stage, severity, and number of wounds. The composition of these calories is critical, as carbohydrates, proteins, and fats each play a unique role in wound repair.

Hydration plays a pivotal role in the wound-healing process, going beyond mere caloric needs. Optimal fluid intake is crucial for maintaining skin turgor, ensuring tissue perfusion, and facilitating oxygen delivery, which are key components in the healing cascade. Water acts as a medium for glucose and micronutrient transport and aids in the elimination of metabolic waste, thereby supporting cellular functions essential for repair. Patients with wounds are particularly vulnerable to dehydration, with risk factors including fever, diarrhea, vomiting, diuresis, fistulae, wound exudate, and inadequate fluid consumption warranting careful assessment. The recommended fluid intake for individuals with wounds is approximately 1 ml/kcal/day, which may need adjustments in response to insensible fluid losses or existing renal or cardiac conditions.^23^, ^24^ Prioritizing water over other fluids is essential, as certain beverages can compromise nutritional status. Alcohol abuse, for instance, is a significant concern. It undermines antioxidant defenses, provokes oxidative stress, and can lead to deficiencies in critical micronutrients like zinc and selenium, which are vital for immune function and wound repair. Chronic alcohol consumption has been linked to decreased plasma levels and increased urinary excretion of zinc, whereas selenium levels have been found to be significantly lower in patients with alcoholic cirrhosis, which improved with supplementation. Additionally, narcotics, whether for medical or recreational use, can induce constipation, nausea, and anorexia, further reducing oral intake and exacerbating nutritional deficits.^25^, ^26^, ^27^

### Macronutrients

Proteins, which are fundamental in introducing indispensable amino acids, avert muscle catabolism and prevent the serious repercussions of protein malnutrition.^28^ Beyond their role in protein synthesis, amino acids are instrumental in maintaining healthy skin. A disruption in amino acid balance can impair protein synthesis within the skin. Essential amino acids are continually lost through the shedding of stratum corneum cells, making protein intake essential to combat skin thinning, dehydration, loss of elasticity, sagging, and wrinkles. During wound healing, proteins are crucial in collagen production.^29^ The ramifications of protein deficiency are profound, leading to compromised fibroblast proliferation and angiogenesis, diminishing collagen synthesis and remodeling.^30^ Proteins are vital for post-surgical wound repair, skin integrity preservation, fluid and electrolyte balance, and facilitating immune response activation. Certain amino acids, like arginine and glutamine, are known to enhance wound healing. Arginine, a precursor to nitric oxide (NO) and proline, is necessary for the inflammatory response, collagen synthesis, and neovascularization, making it a recommended amino acid for accelerating skin wound healing.^31^ The role of glutamine extends to enzymatic, metabolic, antioxidant, and immune responses, offering immunomodulatory and anti-inflammatory effects in wounds.^31^ Glycine also contributes to protein synthesis, wound healing, tissue protection, and immunity.^32^ Protein deficiency hampers wound healing, delaying the transition from the inflammatory to the proliferative phase and reducing angiogenesis and collagen formation, which in turn diminishes fibroblast activities. In chronic wounds, protein needs can drastically increase by up to 250% owing to significant protein loss. Studies have demonstrated the efficacy of protein supplementation in reducing postoperative complications in patients undergoing post-bariatric abdominoplasty, highlighting the importance of protein in enhancing plastic surgery outcomes.^20^, ^33^, ^34^, ^35^, ^36^, ^37^, ^38^

Carbohydrates are pivotal for wound healing as they stimulate production of insulin, a hormone integral to anabolic processes, especially during the proliferative phase of wound healing.^31^ Insulin facilitates glucose uptake into cells, which provides the energy necessary for tissue regeneration. However, maintaining a balance is necessary as hyperglycemia can impair granulocyte function and heighten the risk of infectious complications.^31^ In patients with diabetes mellitus, neuropathic complications can exacerbate wound formation due to reduced pain sensation and consequent neglect of wound care.^31^ Furthermore, factors, such as steroid use, antibiotic therapy, dextrose in intravenous fluids, and physiological stress, that lead to increased cortisol production can contribute to hyperglycemia and complicate the wound-healing process.^39^, ^40^ Fats, particularly essential fatty acids, are vital for cellular membrane integrity and the synthesis of eicosanoids, which are involved in the inflammatory response crucial for wound healing.^41^ Adequate fat intake ensures a well-maintained energy reserve, which is crucial during the prolonged healing process. Moreover, lipids serve as carriers for fat-soluble vitamins, A, D, E, and K, which are involved in cellular differentiation, immune function, antioxidant defense, and coagulation, respectively,—all integral to effective wound healing.^41^

Omega-3 polyunsaturated fatty acids (PUFAs) are integral components of the phospholipid bilayer of cell membranes and play pivotal roles in cellular integrity and metabolic functions.^42^ Eicosapentaenoic acid (EPA), docosahexaenoic acid (DHA), and alpha-linolenic acid constitute the primary omega-3 PUFAs, which act as precursors to eicosanoids, which are signaling molecules that modulate inflammation.^42^ Owing to the inability of the human body to synthesize EPA and DHA, these are typically sourced from marine life, such as fish that consume phytoplankton rich in these fatty acids. Emerging research underscores the facilitative role of omega-3 PUFAs in wound-healing processes.^43^ Their incorporation into the cell membrane is critical during tissue regeneration, particularly following cutaneous injuries. Moreover, omega-3 PUFAs are known to modulate the local inflammatory milieu, potentially expediting the healing trajectory.^43^ A noteworthy investigation revealed that dietary enrichment with omega-3 PUFAs curtailed the synthesis of proinflammatory mediators. In a comparative study, animals fed an omega-3 PUFA-enriched diet demonstrated altered wound-healing dynamics, displaying less robust wounds 30 days post-injury compared to their counterparts on a standard diet.^44^ This paradoxical finding suggests a complex interplay between omega-3 PUFAs and tissue repair mechanisms, necessitating further investigation.^34^ Beyond their role in inflammation and depression amelioration, omega-3 PUFAs are gaining recognition for their potential therapeutic benefits in enhancing recovery following surgical interventions. The multifaceted effects of omega-3 PUFAs on inflammation, immune response, and cellular health position them as significant nutritional considerations in the context of aesthetic procedures and wound management.

### Micronutrients

The role of micronutrients in wound healing has been extensively investigated.^45^^,^^46^ Amino acids such as arginine have emerged as major contributors to the inflammatory process, synthesis of collagen, stimulation of the generation and secretion of growth hormones, and activation of T cells.^47^^,^^48^ Glutamine, another amino acid, reduces infection risks and inflammatory complications by upregulating the expression of heat-shock proteins.^49^ Furthermore, glutamine functions as a precursor to glutathione - an essential co-factor in multiple enzymatic reactions - which contributes to the reinforcement of cell membranes and facilitates amino acid transport across them.^50^ Although the efficacy of arginine is still debatable, the biochemical advantages conferred by amino acids render them an essential consideration in wound-healing and aesthetic procedures.^51^^,^^52^

Vitamins also play a pivotal role in the wound-healing process. Vitamin A deficiency has been shown to impair B and T cell functions and antibody production during inflammation.^31^ It also stimulates the growth of epithelial cells and fibroblasts and is therefore used for dermatological conditions.^21^ Additionally, vitamin A counters the delayed wound healing caused by corticosteroids by down-regulating *TGF-β* and insulin-like growth factor-1 (IGF-1).^53^^,^^54^ Vitamin B compounds, such as thiamine, riboflavin, pyridoxine, and cobalamin, serve as critical cofactors in leukocytic generation, anabolic processes of wound healing, and collagen synthesis.^55^ Consequently, vitamin B deficiency can result in impaired immune function and, thus, an increased risk of infection.^52^^,^^56^^,^^57^ Vitamin C exhibits apparent involvement in collagen synthesis, antioxidant response, and angiogenesis. A systematic review by Thevi et al. advocated using vitamin C for wound healing as it increases recovery rates and leukocyte ascorbic acid levels.^57^, ^58^, ^59^ Emerging evidence of vitamin D deficiency has underscored its potential implication in the wound-healing process.^60^, ^61^, ^62^ A case series by Siregar found that it binds with Vitamin D receptors via calcitriol, regulating the production of several receptors and upregulating the innate immune system while weakening the adaptive immune system.^63^ However, further research is necessary to gain a comprehensive understanding of its complex mechanics and establish a causative or correlative relationship between the two.^62^ In contrast, vitamin E appears to negatively affect collagen synthesis, antioxidant response, and the inflammatory process.^64^ Hobson's 2016 literature review echoed these findings while recommending further research owing to the limited literature regarding this topic. Notably, Leslie et al. 1999 concluded that vitamin E worsened the cosmetic appearance of scars.^65^ Currently, the prevailing consensus highlights the insufficiency of evidence to recommend vitamin E for wound healing.

Minerals have also been implicated in the wound-healing process, attributing to their roles as essential enzymatic components, metalloenzymes, and antioxidants.^31^ Zinc, in particular, promotes re-epithelialization and generation of new tissues.^66^^,^^67^ In inflammation, zinc upregulates the immune response by activating lymphocytes and stimulating antibody production, mitigating the risk of infections.^68^ Copper was found to be involved in all stages of the wound-healing process, due to its numerous roles in several cells, via cytokine and growth factor modulation.^69^ Magnesium similarly promotes wound healing by reducing serum C-reactive protein (CRP) levels and increasing plasma total antioxidant capacity (TAC) concentration.^70^ Razzaghi et al. proposed that a 12-week regimen of magnesium supplements in patients with diabetic foot ulcers yielded advantageous outcomes on ulcer size, glucose metabolism, serum CRP levels, and plasma TAC levels.^70^ Iron, on the other hand, has received less academic scrutiny for its role in wound healing. Its role in oxygen transport in red blood cells has prompted theories that optimal iron levels enable greater tissue perfusion and collagen synthesis.^31^ Iron deficiency has also been correlated with inflammatory diseases such as rheumatoid arthritis and lupus.^71^

### Other nutritional interventions

Beyond the fundamental macronutrients and micronutrients discussed previously, a spectrum of nutritional supplements has been identified to enhance wound healing.^72^ Notably, polyphenolic compounds such as resveratrol (found in berries, peanuts, and red wine) have garnered attention for their therapeutic potential, especially in the context of diabetic foot ulcers (DFUs).^73^ The ability of resveratrol to modulate inflammatory processes via the activation of sirtuins subsequently leads to the reduction of proinflammatory cytokine tumor necrosis factor-alpha and regulation of cellular health.^74^ Similarly, curcumin, a polyphenolic constituent of turmeric, exhibits anti-inflammatory properties by inhibiting the transcription factor NF-κB, effectively reducing inflammation, an essential step in wound-healing promotion.^74^ Additionally, flavonoids, such as naringenin and apigenin, have been shown to mitigate inflammation by increasing the production of nitric oxide via enzymes such as iNOS and COX-2 and inhibiting the release of proinflammatory mediators from BV2 microglia, by acting through NF-κB and the mitogen-activated protein kinase signaling pathway.^75^ Apigenin, another natural flavone, demonstrates protective effects on endothelial cells by reducing apoptosis, primarily by inhibiting caspase 3 activity.^76^

### Association between nutritional status and the risk of postoperative complications

A patient's nutritional status plays a crucial role in their recovery from surgery. Adequate nutrition is vital for optimal wound healing, immune function, and maintenance of muscle strength throughout the postoperative period.^33^^,^^77^^,^^78^ Surgical procedures cause stress on the body that triggers numerous metabolic responses that heighten the body's nutritional requirements.^79^ Preoperative malnutrition can impair the body's ability to heal and cope with the stress of surgery. In this context, malnutrition has been consistently linked with an increased risk of postoperative complications .^80^

Malnourished patients demonstrate higher rates of impaired wound healing, skin breakdown, and wound dehiscence.^2^ Protein malnutrition has been thought to impair collagen synthesis, thus increasing the risk of the aforementioned complications.^81^ Furthermore, malnutrition impairs the immune system function, leading to a higher rate of surgical site infections, which can have serious sequelae such as sepsis.^82^ Moreover, malnutrition is consistently linked to longer hospital stays, which in turn may increase the risk of complications such as venous thromboembolism of hospital-acquired infections such as pneumonia or urinary tract infections.^83^^,^^84^ Overall, several studies have demonstrated that malnourished patients carry a significantly higher risk of postoperative mortality compared with those who are adequately nourished.^85^^,^^86^ Hence, the impact of malnutrition on postoperative outcomes and complications is profound. A summary of the effect of various nutrients on the wound-healing process, along with the complications arising from their deficiencies has been synthesized in Table 1.

**Table 1:** Overview of key nutrients and their specific roles in wound healing and potential complications resulting from their deficiencies.

Nutrient	Role in Wound Healing	Complications from Deficiency
Carbohydrates	Stimulate insulin production that aids in tissue regeneration.	Impaired granulocyte function, increased risk of infection, and exacerbated wound formation in patients with diabetes.
Proteins	Essential for collagen production, immune response activation, and maintenance of skin integrity.	Compromised fibroblast proliferation and angiogenesis, delayed wound healing, and reduced collagen formation.
Fats	Necessary for cell membrane integrity and eicosanoid synthesis.	Poor energy reserve, impaired immune function, and reduced absorption of fat-soluble vitamins.
Vitamin A	Supports growth of epithelial cells and fibroblasts and enhances inflammatory response.	Impaired immune function, delayed wound healing, and reduced collagen synthesis.
Vitamin B	Critical for leukocyte generation, collagen synthesis, and wound healing anabolic processes.	Impaired immune function and increased risk of infection.
Vitamin C	Involved in collagen synthesis, antioxidant response, and angiogenesis.	Delayed wound healing, weakened immune response, and increased risk of scurvy.
Vitamin D	Regulates immune response and receptor production.	Impaired wound healing, increased risk of infection, and weakened adaptive immune system.
Vitamin E	Antioxidant, involved in collagen synthesis and inflammatory response (though evidence of impact on wound healing is mixed).	Potential worsening of scar appearance and impaired collagen synthesis.
Zinc	Promotes re-epithelialization, tissue generation, and immune function.	Delayed wound healing and weakened immune response.
Copper	Involved in all stages of wound healing, modulating cytokines, and growth factors.	Impaired wound healing owing to disrupted enzymatic functions and cytokine modulation.
Magnesium	Reduces serum CRP levels and increases plasma antioxidant capacity.	Delayed wound healing, increased risk of diabetic foot ulcers, and impaired immune function.
Iron	Essential for oxygen transport in red blood cells, facilitates tissue perfusion and collagen synthesis.	Tissue hypoxia, impaired collagen synthesis, and increased risk of inflammatory diseases.
Omega-3 PUFAs	Modulate inflammatory response and critical in cell membrane integration for tissue regeneration.	Altered wound healing dynamics, potentially slower or less robust healing, and reduced anti-inflammatory effects.
Resveratrol	Anti-inflammatory and modulates cytokine activity.	Increased inflammation and potentially delayed wound healing.
Curcumin	Exhibits anti-inflammatory properties by inhibiting NF-κB.	Increased inflammation and delayed wound healing.
Naringenin	Mitigates inflammation and aids in nitric oxide production.	Increased inflammation and potential impairment in wound healing processes.
Apigenin	Reduces apoptosis in endothelial cells and aids in wound healing.	Increased endothelial cell apoptosis and potential delays in wound healing.

### Preoperative nutritional and endocrine optimization

Given the link between malnutrition and operative outcomes, preoperative nutritional optimization for malnourished patients is critical to reduce the associated risks of complications postoperatively. Enhanced recovery after surgery (ERAS) protocols that involve preoperative nutritional optimization have been shown to improve postoperative outcomes and are associated with reduced risk of complications and shorter stays.^87^^,^^88^ Therefore, preoperative assessment and screening of patients is generally recommended to identify patients who are malnourished or at risk of malnutrition where nutritional intervention may be beneficial.

Nutritional assessment is complex and it is important to apply clinical judgment in all cases as no specific malnutrition screening tool will apply perfectly to any individual patient.^89^ Recording the comprehensive history of a patient is the first step in nutritional assessment and involves an understanding of the patient's comorbidities, recent changes in weight or diet, along with other factors that can influence nutritional status such as alcohol use.^89^ Subsequently, anthropometric measurements and body mass index assessments represent moderate objective measurements of nutritional status.^80^ However, fluid shifts can introduce unreliability to body weight calculations and anthropometric measurements vary greatly between individuals.^90^^,^^91^ Biochemical markers such as albumin are sometimes thought to relate to nutritional status; however, in the preoperative surgical patient, albumin levels are influenced by a myriad of factors such as inflammation, capillary permeability, and fluid status.^89^ Clinical scoring tools such as the Subjective Global Assessment or Malnutrition Universal Screening Tool (MUST) can be used to help clinicians make nutritional assessments.^92^ Regardless of the method of assessment, the patient deemed to be malnourished requires preoperative nutritional supplementation to optimize their health prior to surgery.

The timing of preoperative nutritional supplementation will change on a case by case basis; however, most agree that in patients who are malnourished, a 7 to 10 day course of preoperative nutrition is recommended.^80^ Enteral nutrition is typically preferred to parenteral nutrition because it is more physiological, prevents gastrointestinal atrophy, reduces the risk of bacterial translocation and has been associated with reduced incidence of complications such as poor wound healing, postoperative infection and prolonged hospitalization.^93^ Nutritional or energy requirements should be determined, and feeding should be targeted to the patient's specific metabolic needs.^80^^,^^93^ In patients who are unable to eat or reach nutritional requirements through oral intake, nasogastric or nasojejunal feeding can be used. In the appropriate patient, percutaneous endoscopic gastrostomy feeding is also available.^94^ Furthermore, when enteral nutrition is contraindicated, total parenteral nutrition (TPN) can be used.^95^ Throughout the feeding period, patients should be monitored for electrolyte disturbances and complications such as overfeeding, refeeding syndrome, or intravenous line complications in the case of TPN.^91^

Optimizing diabetes management, addressing smoking habits, and balancing endocrine profiles, including testosterone and growth hormone levels, are crucial for effective wound healing and optimal cosmetic outcomes.^96^ In diabetic patients, poorly controlled blood glucose levels can significantly impair wound healing.^96^ Hyperglycemia disrupts normal cellular functions and inflammatory responses, leading to delayed wound closure, increased risk of infection, and poor aesthetic results.^96^ Tight glycemic control is, therefore, essential to enhance the body's natural healing process and reduce complications. Similarly, smoking significantly impedes wound healing and adversely affects cosmetic outcomes due to its impact on tissue oxygenation and cellular function.^97^ The healing process, which involves inflammatory, proliferative, and remodeling stages, relies heavily on proper tissue oxygen levels. Smoking induces tissue hypoxia, a major obstacle to effective wound repair, and increases infection risk post-surgery.^97^^,^^98^ Nicotine, a key component in cigarette smoke, contributes to this issue through its vasoconstrictive action, reducing blood flow and oxygen delivery to the wound. Additionally, carbon monoxide and hydrogen cyanide in smoke further exacerbate tissue hypoxia. Carbon monoxide binds with hemoglobin, limiting oxygen transport, whereas hydrogen cyanide inhibits cellular oxygen metabolism. Beyond these effects, smoking also disrupts essential cellular activities in wound healing. It limits the proliferation of erythrocytes, white blood cells, and fibroblasts, leading to decreased oxygen availability and a weakened immune response. This reduction in immune cell function and collagen synthesis by fibroblasts compromises both the healing process and the strength and appearance of the healed tissue.^97^^,^^98^ Although most research has focused on acute wound healing post-surgery, the negative effects of smoking on chronic wounds, though less studied, are considered similarly harmful. Overall, the evidence strongly suggests that smoking disrupts key aspects of wound healing, leading to slower recovery and poorer cosmetic results. Therefore, it is imperative to rigorously manage diabetes, cease smoking, and ensure optimal preoperative nutrition to significantly enhance wound healing efficiency and achieve superior aesthetic outcomes.

## Conclusion

It is evident that nutrition is influential in wound healing, and some key nutrients extend their benefits to aesthetic outcomes. Several studies have shown associations between nutritional deficits and suboptimal wound healing outcomes. However, the current corpus of evidence remains rather generalized. More observational cohort studies and randomized controlled trials are required to ascertain correlations between various nutrients and their effects on wound healing and aesthetic outcomes.

## Declaration of competing interest

The authors declare no conflict of interest.
